# Spermidine toxicity in *Saccharomyces cerevisiae* due to mitochondrial complex III deficiency

**DOI:** 10.1007/s10522-025-10233-y

**Published:** 2025-04-10

**Authors:** Wei-Hsuan Su, Jessica J. Smith, Evien Cheng, Megan S. Nishitani, Catherine Y. Choi, Kelsey R. Lee, Alexia Pardos Salzano, Samuel E. Schriner

**Affiliations:** School of Pharmacy and Pharmaceutical Sciences, University of California, Irvine, CA USA

**Keywords:** Aging, Yeast, Spermidine, Mitochondrial DNA, Mitochondrial dysfunction

## Abstract

Spermidine is a naturally occurring polyamine present in all cells and is necessary for viability in eukaryotic cells. The cellular levels of spermidine decline as an organism ages, and its supplementation has been found to extend lifespan in yeast, worms, flies, mice, and human cultured cells. The lifespan extending effect of spermidine is thought to be due to its ability to induce autophagy, a turnover of cellular components. Mitochondrial dysfunction is believed to be a major driver of the aging process. We asked whether spermidine could rescue mitochondrial dysfunction using the yeast *Saccharomyces cerevisiae* lacking mtDNA (ρ^0^ cells) as a model. Not only was spermidine unable to rescue survival in ρ^0^ cells, but it appeared to exhibit toxicity resulting in a shortened lifespan. This toxicity appears to not be due to the loss of mitochondrial respiration, elevated oxidative stress, or depleted ATP. Spermidine toxicity could be recapitulated by the genetic or pharmacological inactivation of mitochondrial complex III. It can also be prevented by the impairment of autophagy, through the inactivation of *ATG8*, or by impairment of mitochondrial complex II through the inactivation of *SDH2*. Spermidine toxicity in ρ^0^ cells was present in yeast strains BY4741 and W303, but not D273-10B, demonstrating genetic variance in the phenotype. Thus, caution may be suggested regarding the use of spermidine to alleviate aging in humans. Depending on the genotype of the individual, spermidine could potentially harm the very individuals it is intended to help.

**Graphical abstract:**

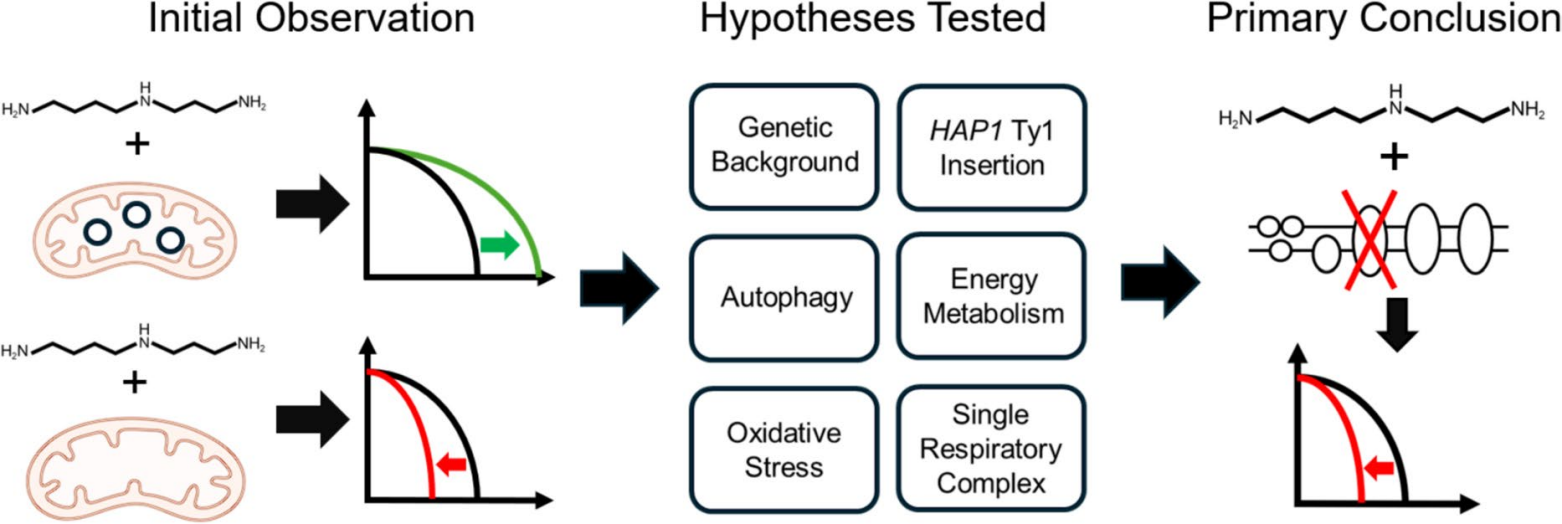

**Supplementary Information:**

The online version contains supplementary material available at 10.1007/s10522-025-10233-y.

## Introduction

The discovery of genes that could extend lifespan in model organisms has spurred a search for small molecules and dietary regimes that could do the same (de Cabo et al. 2014). Most of these are thought to have a causal relationship to dietary restriction, mitochondrial function, and oxidative stress (de Cabo et al. 2014). Spermidine (SPD) is one such compound and is thought to act through the induction of autophagy, which is a process leading to the turnover of cellular components (Madeo et al. 2018; Eisenberg et al. 2009). Supplementation of SPD has been shown to extend lifespan in yeast, worms, flies, mice, and human culture cells (Eisenberg et al. 2009, 2016), while genetic impairment of autophagy prevented such lifespan extension in worms and flies (Eisenberg et al. 2009). Autophagy has also been proposed to be one of the mechanistic explanations for the positive effects of dietary restriction (Chung and Chung 2019). SPD is present in all cells and appears to be necessary for survival in eukaryotic cells (Igarashi and Kashiwagi 2019). The complete role of SPD in the cell is not exactly clear, though in addition to autophagy, it is thought to be able to stabilize RNA, particularly tRNAs, by exchanging positions with Mg_2_^+^ ions, and may have important functions in DNA metabolism (Igarashi and Kashiwagi 2019).

Mitochondria are essential components of eukaryotic cells where they produce ATP, shuttle low-energy electrons to oxygen, generate reactive oxygen species, store calcium ions, metabolize fatty acids, and regulate cellular growth, differentiation, and death (Harrington et al. 2023). The DNA present in the mitochondria lacks many of the protective proteins and repair systems present in nuclear DNA, making them especially vulnerable to damage and mutation (Nadalutti et al. 2022). This is particularly problematic due to the proximity of reactive oxygen species generated by the respiratory chain. Mutations in mitochondrial DNA (mtDNA) cause severe early life diseases, and mitochondrial dysfunction is believed to be involved in many late life disorders and may be a driver of the aging process itself (Bratic and Larsson 2013; Miwa et al. 2022). The importance of mitochondrial function in aging and disease has been demonstrated by the beneficial effects of mitochondrial-targeted antioxidants. For example, the small molecule MitoQ has been shown to inhibit memory loss and neuropathy, and improve survival in Alzheimer’s disease model mice (Young and Franklin 2019). The peptide-based SS-31 has been shown to improve cardiac function in aged mice (Chiao et al. 2020). Mice expressing the antioxidant enzyme catalase targeted to the mitochondria not only exhibited an increased mean and maximum lifespan, but also improved parameters of cataract formation, insulin sensitivity, exercise performance, cardiac pathology, and invasive breast cancer (Schriner et al. 2005; Wolf et al. 2005; Lee et al. 2010) (Li et al. 2009) (Chiao et al. 2020; Goh et al. 2011).

In this study, we used the yeast *Saccharomyces cerevisiae* to ask whether SPD was dependent on functional mitochondria to extend lifespan. This question could also be phrased as to whether SPD could rescue mitochondrial dysfunction. Aging in yeast can be evaluated in two different replicative lifespan and chronological lifespan. Replicative lifespan is generally interpreted to represent replicating cells in the animal, such as intestinal epithelium (Longo et al. 2012). Whereas chronological lifespan is considered to be more representative of post mitotic cells such as neurons and cardiomyocytes (Longo et al. 2012). Due to the prevalence of neurovegetative and cardiovascular disease in humans, we focused on the chronological model in this study. In chronological aging, yeast are allowed to consume all of the glucose the media, after which they arrest. Lifespan is defined by how long yeast survive once arrested. Surprisingly, we found that SPD was toxic in some strains lacking mtDNA. This study raises an important issue: if mitochondrial dysfunction were a true driver of many diseases and the aging process itself, then the use of SPD as a treatment for aging and age-related diseases could potentially be harmful in some individuals.

## Materials and methods

### Yeast and media

All yeast media components were purchased from Fisher Scientific (Hampton, NH) except where indicated. Yeast extract-peptone-dextrose (YPD) media was made with 10 g/L yeast extract (Fisher Scientific,), 20 g/L peptone, and 20 g/L dextrose. Yeast extract-peptone-glycerol (YPG) media was made with 10 g/L yeast extract, 20 g/L peptone and 2% glycerol. Synthetic complete (SC) media and SC dropout media was made with 28.7 g/L powder (Sunrise Science, Knoxville, TN). Tetracycline (Sigma-Aldrich, St. Louis, MO) was diluted to a concentration of 10 mg/mL with 70% EtOH solution and used at a final concentration of 10 μg/mL in all cultures to prevent bacterial contamination. SPD was added to media at a final concentration of 4 mM. The YPD plates were made with the addition of 2% Bacto Agar. To select for KanMX, G418 (Gold Bio, Olivette, MO) was added to media at a final concentration of 200 μg/mL. To inhibit complex III, antimycin A was added to cultures at a final concentration of 5 μM.

Yeast strains used in this work are listed in supplementary Table S1. The double knockout of *NDE1* and *NDE2* was generated by CRISPR/Cas9 homology directed repair by targeting *URA3MX* to *NDE2* in the *nde1Δ::KanMX* background. Repair of *hap1* was conducted by CRISPR/Cas9 homology directed repair by using a wildtype *HAP1* repair template. Guide RNA and repair template sequences are listed in supplementary Table S2. Generation of ρ^0^ cells was accomplished by the selective elimination of mtDNA by treatment with ethidium bromide (Slonimski et al. 1968). Ten μL of yeast was added to 50 mL YPD along with 50 μL of a 10 mg/mL EtBr solution, and then grown to saturation. For both ρ^0^ cells and *CYT1-*, *COX6-*, and *ATP1-*deficient cells (Fig S1), loss of respiration was verified by the inability to grow on glycerol.

The repair of *HAP1* and generation of the *NDE1*, *NDE2* double knockout was performed using CRISPR/Cas9, and the plasmids pML104 and pML107 were gifts from Dr. John Wyrick (Addgene plasmid #67,639). The guide RNAs for *HAP1* and *NDE2* were designed using the CRISPR tool set by Dr. John Wyrick. The URA3MX repair templates were generated by Twist Bioscience. Targeting of the *HAP1* and *NDE2* genes were selected by growth in SC-ura media for HAP1R BY4741 and SC-ura -leu for NDE1, NDE2 double Knockout BY4741. Successful targeting was verified by sequencing (Azenta, Burlington, MA).

### Chronological lifespan (CLS) assays

The CLS assays were initiated by the addition of 10 μL of the respective yeast strains to 50 mL of the appropriate media and grown to saturation with an OD_600_ of 1.3 at which time constitutes day 0. On day 0 and every other day thereafter, yeast were serially diluted and plated on fresh media and grown for two days. Colonies were then counted and the proportion alive was calculated by dividing the surviving number for each day by the number of cells on day 0. In our initial study, we defined lifespan at the point of 99% mortality. However, we found that 90% mortality was sufficient to statistically determine any effect on survival. To minimize any potential confounding effect due to the growth advantage in stationary phase (GASP) phenomenon (Zambrano and Kolter 1996), we further elected to end our lifespan assays at 90% mortality.

### Gene expression and mtDNA content

Yeast cultures were grown in 50 mL of YPD media with 50 μL tetracycline for two days at 30 °C until saturation was reached (an OD_600_ of 1.3), at which point SPD was added to the experimental cultures to a final concentration of 4 mM. The expression levels of *ATG8* and *ATG33* were measured on day three of growth using RT-PCR. Relative expression levels were estimated by comparing the Ct values of *ATG8* and *ATG33* to those of the housekeeping gene *ACT1*. RNA from each culture was extracted using the YeaStar RNA kit (Zymo Research, Tustin, CA) per the manufacturer’s instructions. For each extraction, 1 mL of culture was used and eluted in 60 μL nuclease-free water. RNA was reverse transcribed into cDNA using the LunaScript 5 × RT supermix (New England Bio Labs, Ipswich, MA). Each reaction contained 1 μL RNA, 4 μL 5 × LunaScript RT supermix, and 15 μL Nuclease-free water. The thermal cycler conditions were set as follows: 25 °C for 5 min, 46 °C for 20 min, 95 °C for 1 min, 4 °C hold. The amplified cDNA samples were analyzed by RT-PCR using the Bio-Rad MiniOpticon. In order to prepare samples, 1 μL of 1 uM forward and reverse primers, 20 μL ultra-pure water, and 23 μL iTaq Universal SYBR Green Supermix were mixed using a pipette to avoid creating bubbles. One μL of cDNA and 19 μL of the super master mix were used in each well. The forward and reverse primers were designed with primer III and the primers were synthesized by Integrated DNA Technologies (Coralville, IA). The primers had an initial concentration of 100 μM and were diluted to 1 μM using ultra-pure water for the experiment. PCR thermal cycler conditions were set as follows: hot start at 95 °C for 30 s, PCR cycle of 45 cycles of 95 °C for 15 s and 50 °C for 30 s. For mtDNA content, total DNA was extracted at mid-log phase using the YeaStar extraction kit. The number of mtDNA copies per cell was calculated by dividing the Ct value of a mtDNA target (*COX1*) amplification by the Ct value of a nuclear target (*GAL1*) for each sample of DNA.

### ATP Assay

Ten μL of yeast were added to 50 mL YPD media with 50 μL tetracycline and grown until saturation was reached (approximate OD_600_ of 1.3). SPD was then added to a final concentration of 4 mM. The ATP standard curve was prepared with 1 mM, 100 nM, 10 nM, 1 nM, and 0 nM of ATP. For the standard curve, 100 μL of each dilution (and one for water) was added to individual wells respectively in duplicate in a white 96-well plate. To measure steady-state ATP levels, yeast were counted on a hemocytometer and diluted to 10^6^ cells/mL, and 100 μL of respective yeast cultures were pipetted into each individual well in duplicate. To every well, 100 μL of BacTiter-Glo (Promega, Madison, WI) was added. The plate was incubated for 1 min at 30 °C. Luminescence was measured using BioTek Synergy HT microplate reader and the subsequent cellular ATP concentrations were calculated from the standard curve.

### Citrate synthase assay

Yeast cultures were grown in 50 mL of YPD media with 50 μL tetracycline for two days at 30 °C until saturated and SPD was added to the experimental cultures to reach a concentration of 4 mM. Yeast were pelleted by centrifugation at 10,000 g for 2 min, washed in 50 mM Tris–HCl, vortexed with glass beads 5 times for 30 s, and placed on ice for 30 s between vortexing. Yeast and beads were centrifuged at 10,000 g for 2 min and supernatants were used as samples in the citrate synthase assay. The reaction buffer was prepared with 50 mM Tris–HCl (pH 8.0), 0.3 mM Acetyl CoA, and 0.1 mM 5,5'-dithiobis(2-nitrobenzoic acid (Sigma-Aldrich, St. Louis MO). Five hundred forty μL of the reaction buffer was added into each yeast extract sample. Thirty μL of the yeast extract supernatant was added to the corresponding yeast extract sample. The sample was vortexed and incubated at 30 °C for 5 min. In a 96-well plate, 10 μL of oxaloacetic acid and 190 μL mix (buffer and extract) was added per well for each duplicate sample. A blank was prepared with 540 μL of buffer and 30 μL of H_2_O. The plate was read for 5 min at 412 nm. A Bradford assay was performed to normalize the citrate synthase concentration in the total protein concentration using a standard curve generated with bovine serum albumin (Sigma-Aldrich, St. Louis MO).

### Measurement of media pH

Yeast cultures were grown in 50 mL of YPD media with 50 uL tetracycline for two days at 30 °C until saturation was reached. SPD was added to the experimental cultures to a final concentration of 4 mM. Ten mL of each yeast sample was taken and measured for its pH with an OHAUS pH meter.

### Paraquat survival assay

Yeast were grown to saturation in 50 mL of YPD media with 50 μL tetracycline, and then SPD was added to a final concentration of 4 mM. After 24 h, yeast were serially diluted to 10^6^, 10^5^, 10^4^, 10^3^, and 10^2^ cells/mL and incubated with 1.25 mM of paraquat (Sigma-Aldrich, St. Louis MO). Ten μL of each dilution was spotted onto YPD + Tet plates. The plates were incubated at 30 °C for 2 days and subsequently photographed and analyzed.

### Statistical analyses

Data was analyzed using GraphPad Prism (Dotmatics, Boston, MA). All results are presented as means ± SEM. Unless otherwise indicated, sample sizes for all assays are six independent cultures. The statistical tests used are indicated in the figure captions. *P*-values less than 0.05 were considered to be statistically significant.

## Results

### Spermidine shortens lifespan in ρ^0^ BY4741 yeast and closely related strains

**Fig. 1:**
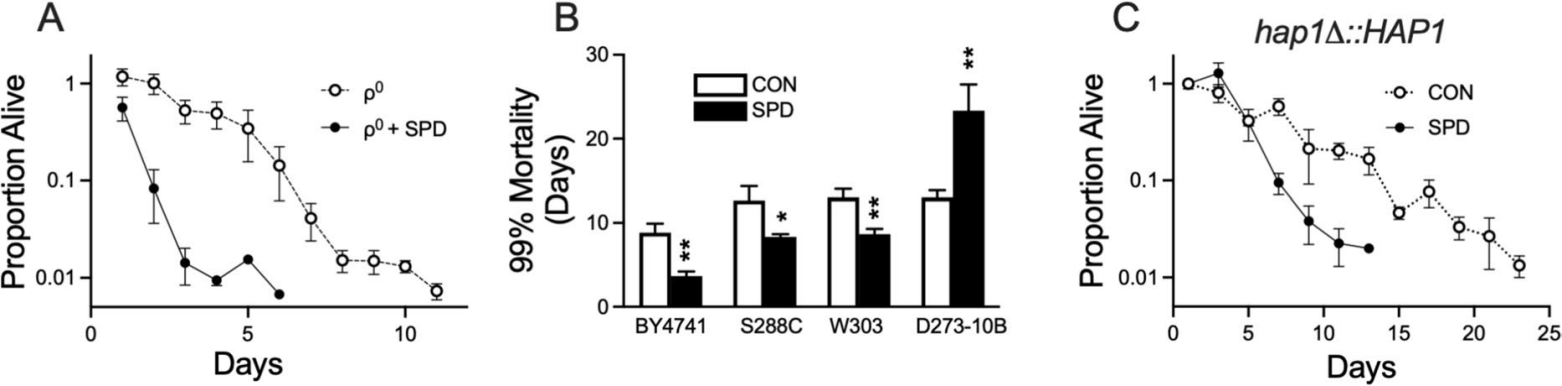
The effect of SPD on **A** survival of BY4741 ρ^0^ cells, **B** 99% mortality lifespan of BY4741, S288C, W303, and D273-10B ρ^0^ cells, and **C** survival of BY4741 ρ^0^ cells with *hap1* repaired to wildtype (HAP1R). N = 6 independent cultures for all groups except for later data points in the survival assays where individual cultures gradually reach their end point. *P < 0.05, **P < 0.01, ****P < 0.0001, unpaired t test vs. respective control

Mitochondrial dysfunction is thought to play a significant role in aging (Bratic and Larsson 2013), and the polyamine SPD has been shown to extend lifespan in model systems (Eisenberg et al. 2009). Given these two observations, we asked whether SPD could rescue mitochondrial dysfunction. We tested this idea in the yeast *Saccharomyces cerevisiae* devoid of mitochondrial DNA (mtDNA). Yeast deficient in mtDNA (termed ρ^0^ cells) were generated by treatment with ethidium bromide which preferentially causes cells to lose their mtDNA. To our surprise, not only was SPD unable to extend chronological lifespan in yeast, but it also appeared to be toxic, resulting in a shortened lifespan relative to untreated ρ^0^ cells (Fig. 1A). We then asked whether this was a general phenomenon or specific to individual yeast strains, such as BY4741, the strain we initially used. Toxicity was evident in the isogenic S288C strain and in W303 which shares 85% of its genome with S288C (Ralser et al. 2012). However, SPD was able to extend lifespan in the more distantly related D273-10B strain (Fig. 1B). BY4741 is known to have a high petite frequency, which is a result of the loss of functional mitochondrial respiration leading to slow growth and small colony formation (Whittaker 1979; Lasserre et al. 2015). Deletion of the transcription factor heme activator protein 1 (HAP1) has been shown to elevate petite formation (Mattoon et al. 1990). BY4741 contains an insertion of a TY transposon in the carboxy end of the protein (Pfeifer et al. 1989). We used CRISPR/Cas9, and homology directed repair to return HAP1 to its wildtype sequence. We found that SPD still exhibited toxicity in BY4741 with the repaired *HAP1*, ruling out *HAP1* as a potential explanation (Fig. 1C).

### Spermidine toxicity in ρ^0^ yeast is related to autophagy

**Fig. 2:**
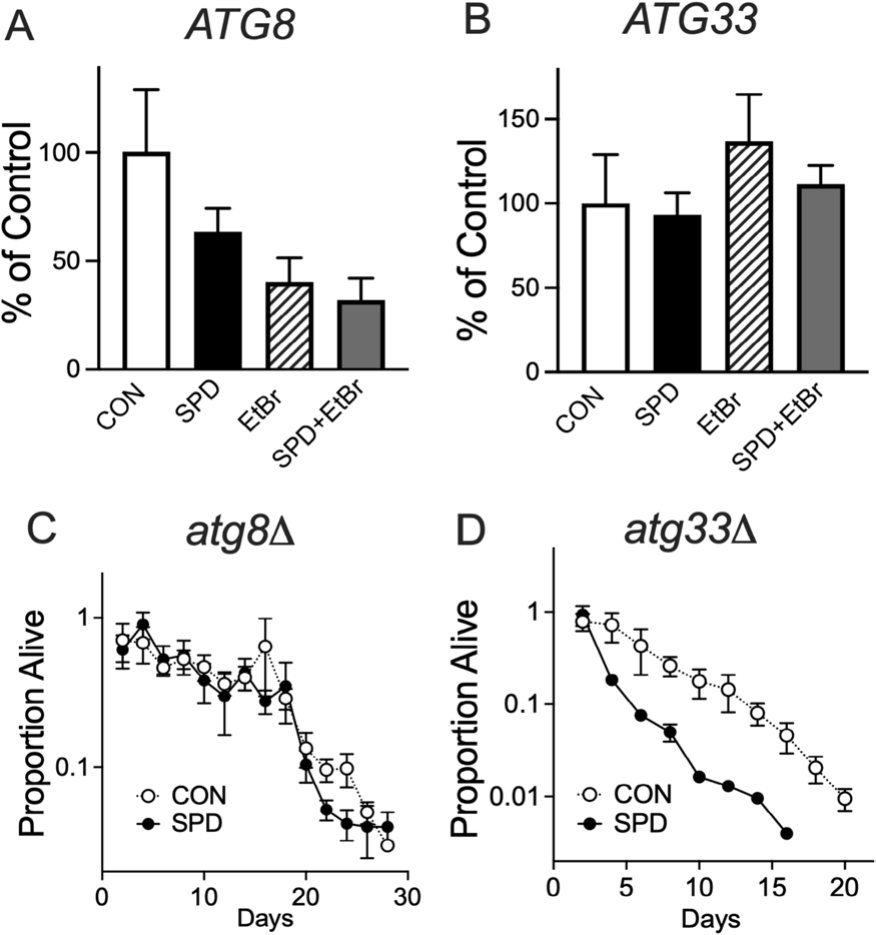
Expression levels and effect on survival of autophagy related genes. **A** Down-regulation of *ATG8* by EtBr, *P* < 0.05, two-way ANOVA **B** *ATG33* expression unaffected by either SPD or EtBr *P* > 0.05, two-way ANOVA. **C** Loss of *ATG8* had no effect on lifespan with regard to SPD, whereas **D** SPD still shortened lifespan in cells deficient in *ATG33*, *P* < 0.005 for median lifespan, t test. N = 6 independent cultures for all groups except for later data points in the survival assays where individual cultures gradually die off

We then hypothesized that the spermidine toxicity may have resulted from the induction of autophagy, which requires ATP. This could result in ATP levels low enough to compromise survival in ρ^0^ cells. We asked whether SPD and EtBr combined could induced expression of autophagy-related genes. We found that both SPD and EtBr appeared to down-regulate *ATG8*, (Fig. 2A) but had no effect on the mitophagy-specific *ATG33* (Kanki et al. 2009) (Fig. 2B). We then asked whether the toxicity we observe is dependent on autophagy-related proteins. Deficiency of *ATG8* eliminated SPD toxicity in ρ^0^ cells (Fig. 2C and Supplementary Fig S1B), whereas spermidine still exhibited toxicity in these cells with the loss of *ATG33* (Fig. 2D and Supplementary Fig S1C).

### The effect of spermidine on ATP levels, metabolism, and mitochondrial content

**Fig. 3:**
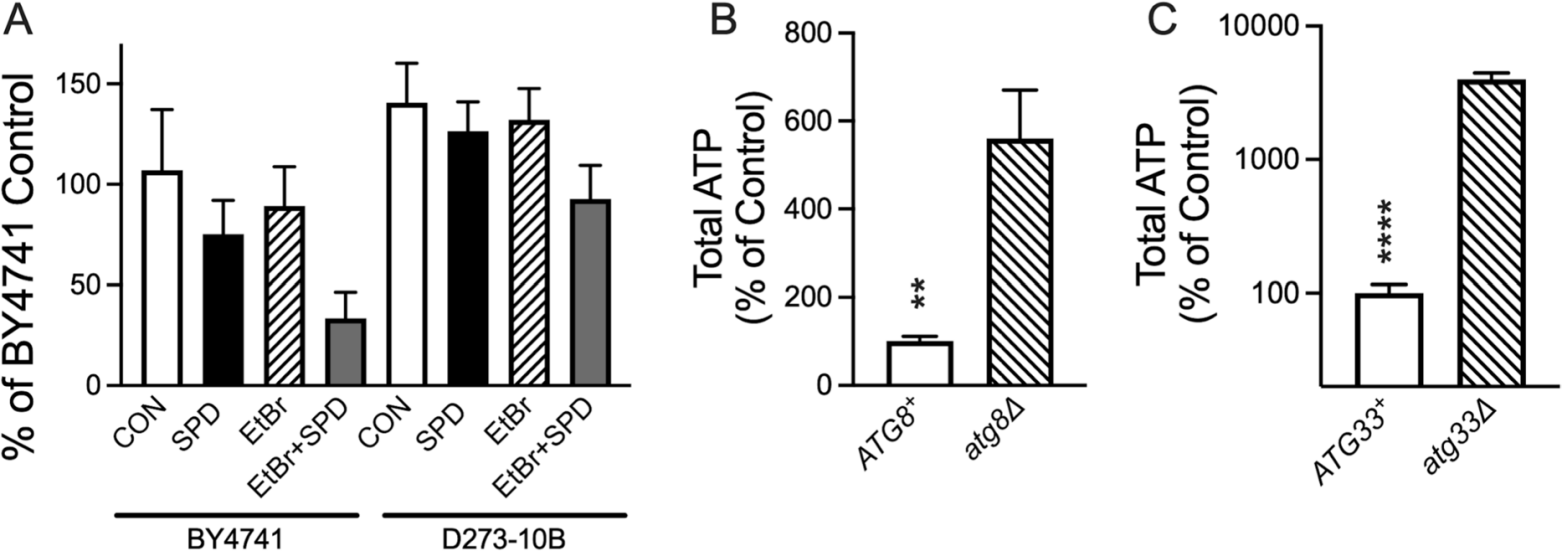
Steady-state yeast ATP levels. **A** ATP levels were impacted by both SPD (*P* < 0.05) and yeast strain (*P* < 0.005), three-way ANOVA. Impairment of autophagy increases ATP levels in **B** *ATG8* deficient cells (*P* < 0.001, t test vs. KO) and radically increases ATP in **C** *ATG33* deficient cells (*P* < 0.00001, t test vs. KO). Note: The Y axis for panel C is on a log scale. n = 6 for each group

**Fig. 4:**
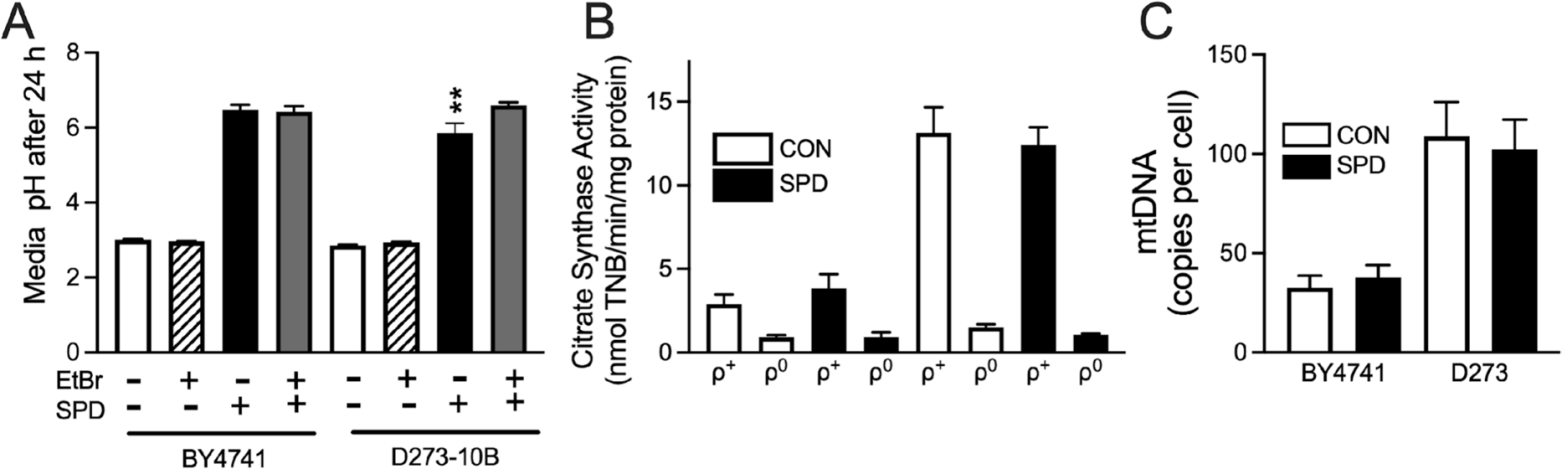
The effect of SPD on mitochondrial content and overall metabolism. **A** When given SPD, D273-10B ρ^0^ cells acidify media to a greater extent to D273-10B ρ^+^ cells (*P* < 0.005) or BY4741 ρ^+^ or ρ^0^ cells (*P* < 0.05), Tukey’s multiple comparison test. n = 5–7 for mtDNA content n = 6 for all other groups. **B** Mitochondrial content via measurement of citrate synthase was markedly higher in both ρ^+^ and ρ^0^ D273-10B cells relative BY4741 cells. However, SPD had no effect on either parameter in either strain (P = 0.92, three-way ANOVA for SPD). **C** D273-10B cells had a much higher mtDNA content relative to BY4741 cells (*P* < 0.0001, two-way ANOVA), but again, SPD had no effect (*P* = 0.96, two-way ANOVA)

The shortened lifespan seen in SPD treated ρ^0^ cells could be a simple result of insufficient ATP levels. That is, SPD drives the consumption of ATP through the induction of autophagy in cells that are already energy depleted due to impaired respiration. We found that SPD did indeed further decrease ATP levels in ρ^0^ cells compared to ρ^+^ cells (Fig. 3A) in both BY4741 and D273-10B strains. However, consistent with this possibility, ATP levels were significantly lower in BY4741 compared to D273-10B. We then examined ATP levels in *ATG8-* and *ATG33*-deficient cells and surprisingly found significant increases in SPD-fed cells compared to controls (Fig. 3B and C). Deficiency in *ATG33* resulted in a greater than 50-fold increase in ATP levels. Considering that SPD shortened lifespan in *ATG33*-deficient cells, it is unlikely that low ATP levels could be the driving factor for spermidine toxicity in ρ^0^ cells. Yeast consume glucose and produce ethanol, which they then convert into acetic acid. Therefore, acidification of the media can be used as a simple marker of overall metabolism. SPD contains three amines with pKa values significantly above neutral, resulting in a marked increase in the pH of the media. As acidification of the media may limit yeast chronological lifespan (Mirisola and Longo 2012), it could be possible that SPD extends lifespan through the trivial mechanism of buffering pH. This is unlikely to be true due to the toxicity of SPD that we see while pH is still being maintained. Despite this confounding factor, a significant 3-way interaction between SPD, EtBr, and strain was found. This was visualized as a decrease in pH in D273-10B cells fed SPD relative to the same cells lacking mtDNA (Fig. 4A). Thus, there is some metabolic function in D273-10B cells that can be inhibited by SPD and EtBr that is not present in BY4741 cells. We then asked whether SPD had a negative effect on total mitochondrial content and on mtDNA levels. While we found that D273-10B cells had much higher levels of both compared to BY4741 cells, SPD had no effect on either parameter (Fig. 4B and C).

### Spermidine and oxidative stress

**Fig. 5:**
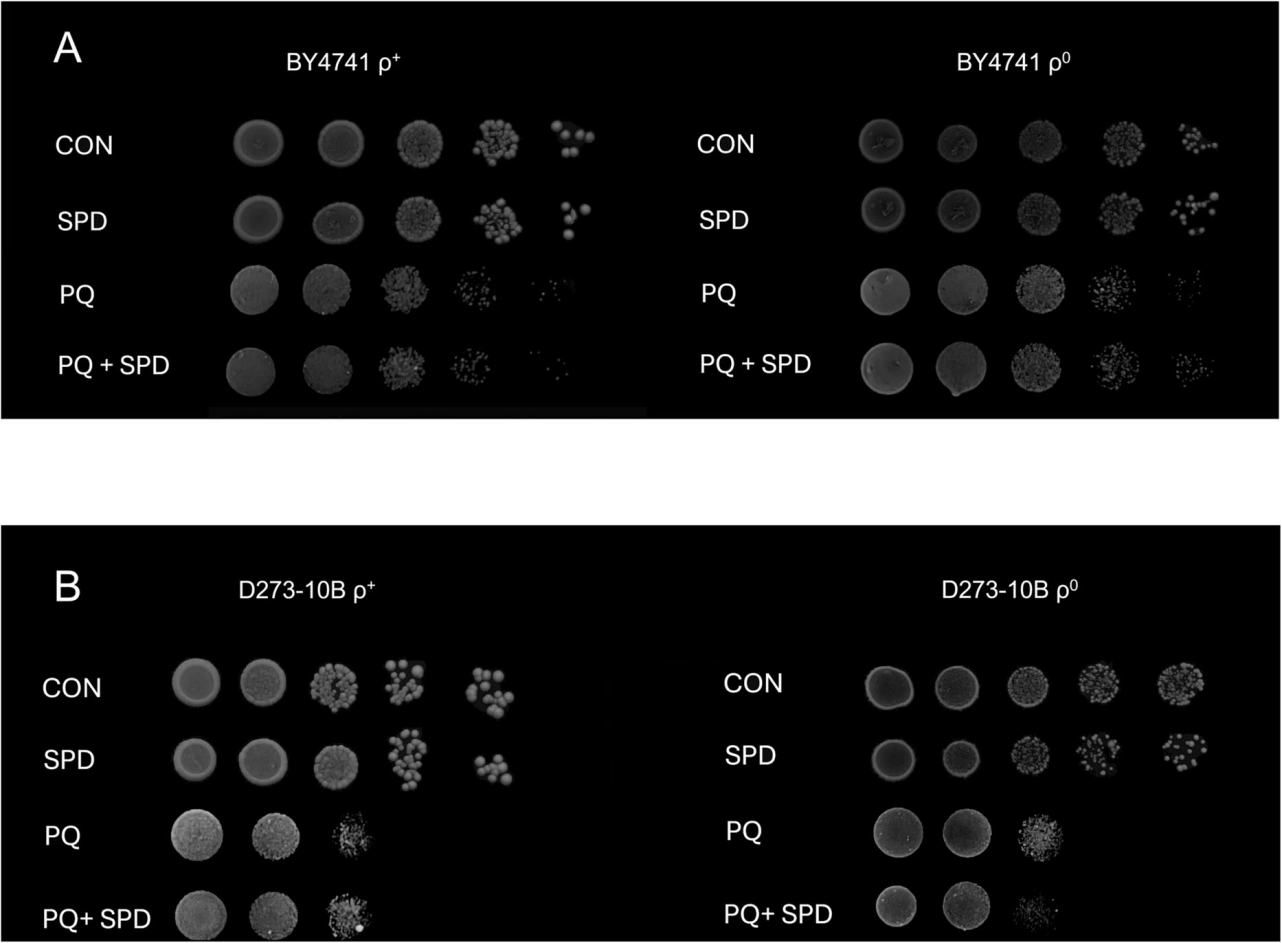
Resistance to PQ by SPD supplementation.** A** PQ tolerance in control and SPD fed ρ^+^ and ρ^0^ BY4741 cells. **B** PQ tolerance in control and SPD fed ρ^+^ and ρ^0^ D273-10B cells

SPD has been proposed to cause toxicity through the generation of superoxide radicals (O_2_^−^) (Kumar et al. 2022). Therefore, we hypothesized that perhaps the toxicity we see in ρ^0^ cells is due to elevated sensitivity to O_2_^−^. We then tested the tolerance of BY4741 and D273-10B ρ^+^ and ρ^0^ cells to O_2_^−^ insult using paraquat, a cellular generator of O_2_^−^. BY4741 ρ^0^ cells appeared to better tolerate paraquat than did ρ^+^ cells, and in either case, SPD had no effect (Fig. 5A). A different picture emerged for D273-10B. Both ρ^+^ and ρ^0^ control cells had similar responses to paraquat, however SPD seemed to sensitize ρ^0^ cells (Fig. 5B).

### Impaired mitochondrial complex III alone results in spermidine toxicity

**Fig. 6:**
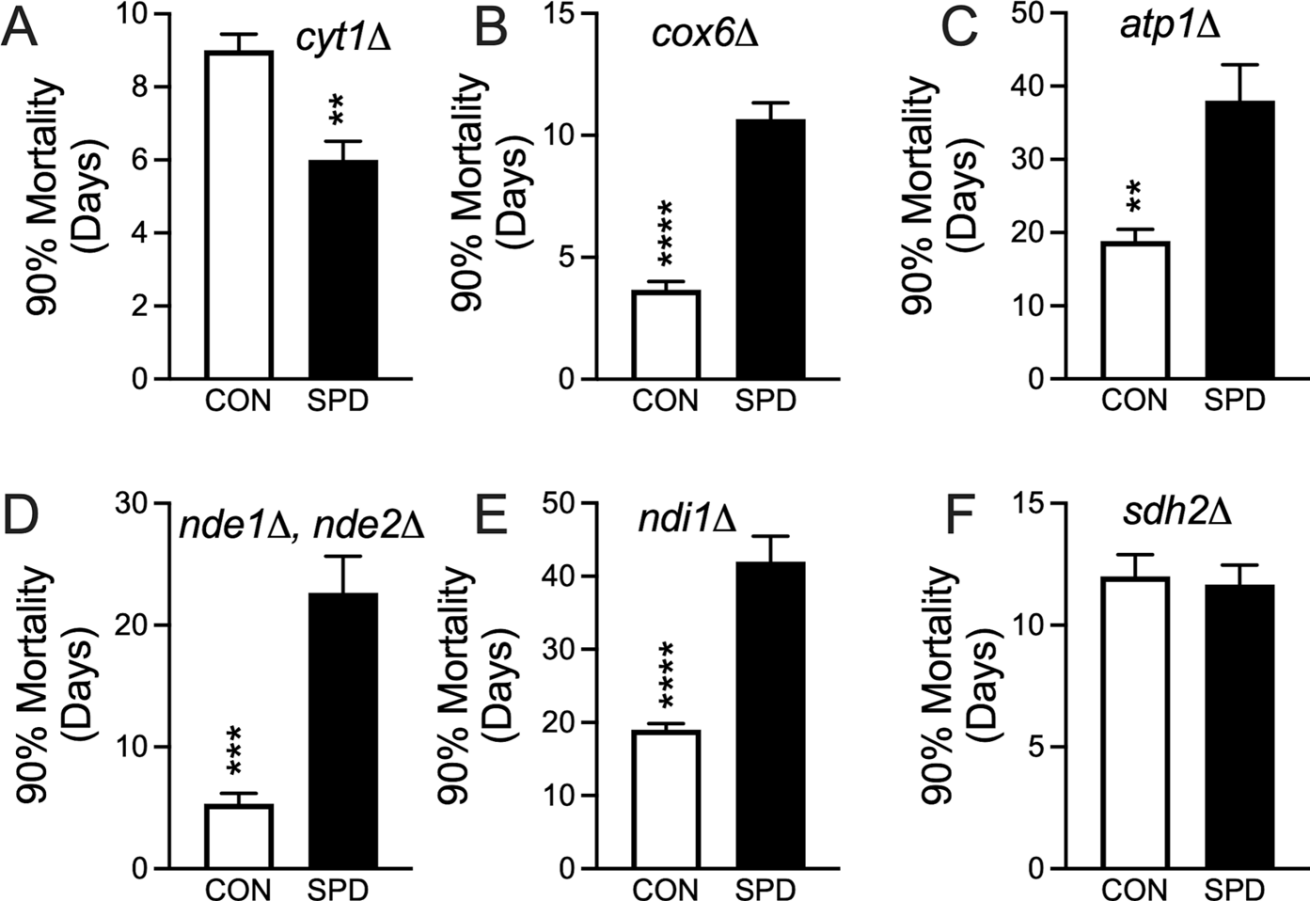
Lifespan 90% mortality for single/double gene knockouts of nuclear encoded respiratory chain complex subunits. SPD increased lifespan when fed to **A** decreased lifespan in cells lacking *CYT1* (*P* < 0.005). SPD increased lifespan in cells deficient in either **B** *COX6* (*P* < 0.0001) or **C** *ATP1* (*P* < 0.01), **D** cells deficient in both *NDE1* and *NDE2 (P* < 0.001) and **E** *NDI1* (*P* < 0.0001). SPD had **F** no effect on cells lacking *SDH2* (*P* = 0.78) and t test. N = 6 independent cultures for all groups except for later data points in the survival assays where individual cultures gradually die off

**Fig. 7:**
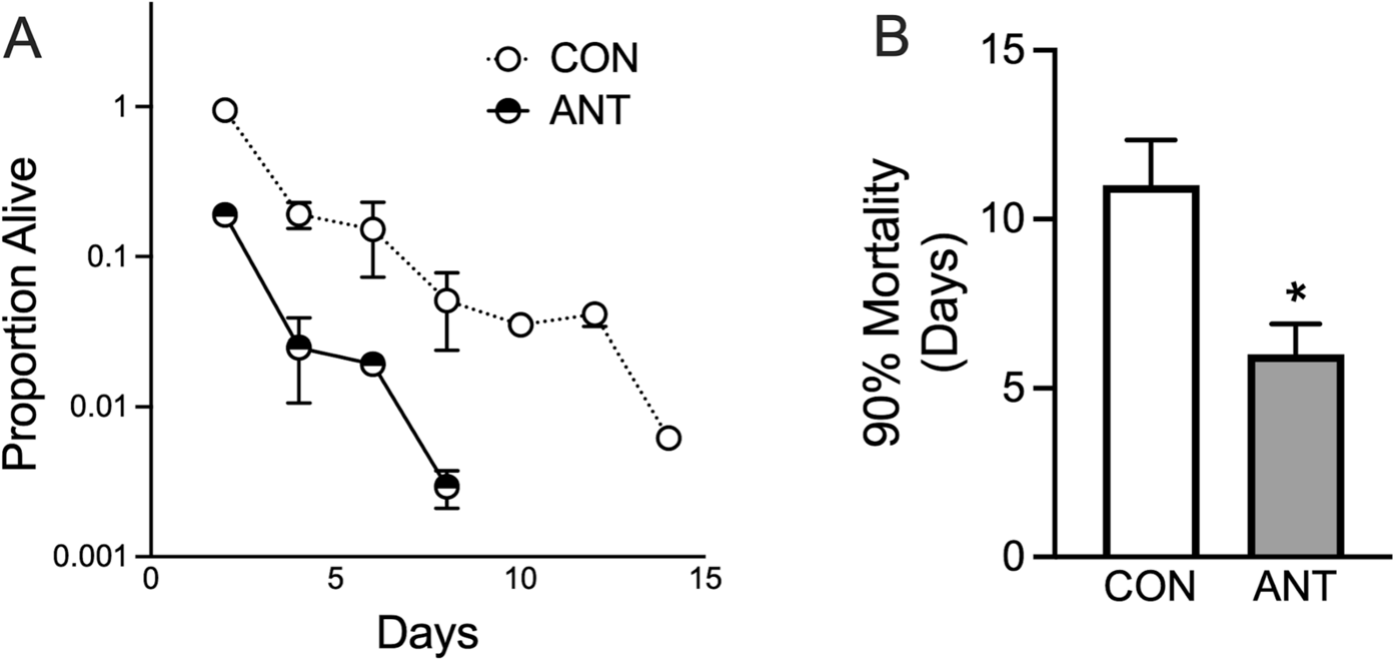
The effect of antimycin A (ANT) in BY4741 ρ^+^ cells on **A** survival and **B** 90% mortality lifespan. *P < 0.05, t test. N = 6 independent cultures for both groups except for later data points in the survival assays where individual cultures gradually die off

The *S. cerevisiae* mitochondrial genome encodes subunits of the respiratory chain complexes III, IV, and V (Dujon 2020). Because EtBr treatment removes the entire mtDNA circle, all three complexes are disrupted. We wished to determine whether the disruption of individual complexes one-by-one would be sufficient to cause SPD toxicity. This was the case for deficiency in CYT1 (Fig. 6A), demonstrating that disruption of complex III alone could recapitulate the SPD toxicity. However, surprisingly SPD was able to still extend lifespan in cells deficient for COX6 or ATP1 (Fig. 6B and C), the lack of which impair complexes IV and V, respectively. All three mutants are unable to grow on glycerol (supplementary Fig S2) showing that each individual mutant was able to disable respiration. While not encoded by mtDNA, we wished to determine whether the loss of other respiratory chain proteins could result in SPD toxicity. In most eukaryotes, electrons predominantly enter the respiratory chain via NADH through the mitochondrial complex I, which is quite large and composed by up to 45 subunits (Hirst 2013). In the case of *S. cerevisiae*, such a complex is absent, and replaced by three individual NADH dehydrogenases, Nde1p, Nde2p, and Ndi1p. Nde1p and Nde2p are known to be paralogs, (Luttik et al. 1998) so we assayed yeast with both of their genes deleted. We found that SPD was still able to extend lifespan when these NADH dehydrogenases were deleted (Fig. 6D and E). Electrons also enter the respiratory chain though complex II (succinate dehydrogenase) via FADH_2_ and the tricarboxylic acid cycle (Baile and Claypool 2013). Interestingly, SPD was no longer toxic in *sdh2*-deficient cells but was not able to extend lifespan either (Fig. 6F). We used the complex III inhibitor antimycin A as a pharmacological means to verify the role of complex III in creating this SPD toxicity. As in *cyt1*-deficient cells, antimycin A was also able to create this phenotype (Fig. 7A and B).

## Discussion

Over the years, several compounds have been identified that are able to extend the lifespan of yeast, worms, flies, and mice in the laboratory. This includes dinitrophenol, metformin, resveratrol, rapamycin, and spermidine (Barros et al. 2004; Caldeira da Silva et al. 2008; Kazi et al. 2017 Martin-Montalvo et al. 2013; Choi et al. 2013; Bjedov et al. 2010; Harrison et al. 2009; Howitz et al. 2003; Eisenberg et al. 2016). At the same time, mitochondrial dysfunction is thought to be one of the main drivers of the aging process (Bratic and Larsson 2013). Using the yeast *Saccharomyces cerevisiae*, we asked whether these compounds could extend chronological lifespan in a model of mitochondrial dysfunction. As such a model, we cured the yeast of mitochondrial DNA using ethidium bromide. We found that dinitrophenol, metformin, rapamycin, and resveratrol were not able to extend lifespan in absence of mtDNA (unpublished data). Yet, we found spermidine to be toxic in ρ^0^ cells. We then wished to further explore the source of this toxicity. Spermidine is a naturally occurring polyamine present in all cells and necessary for viability in eukaryotic cells (Igarashi and Kashiwagi 2019). The levels of SPD decline naturally as an organism ages, and its supplementation has been found to extend lifespan in yeast, worms, flies, mice, and human cultured cells (Eisenberg et al. 2009, 2016). We therefore expected that SPD would be able to extend lifespan in cells exhibiting mitochondrial dysfunction. Using the yeast *Saccharomyces cerevisiae* lacking mtDNA (ρ^0^ cells) as a model for mitochondrial dysfunction, we surprisingly found that SPD shortened chronological lifespan.

We initially selected the yeast strain BY4741 for our study as it is commonly used, contains convenient selectable markers, and knockouts of nearly all nonessential genes are commercially available. However, some consider BY4741, and its parental strain S288C, to be a poor choice for mitochondrial research as there are high frequencies of petite formation due to spontaneous occurrence of cells deficient in respiration (Dimitrov et al. 2009). The presence of a transposon disrupting the 3’ end of the transcription factor heme activator protein 1 (HAP1) is thought to contribute to this (Mattoon et al. 1990). We immediately considered this disrupted *hap1* to be a potential cause of the SPD toxicity we see. Using CRISPR/Cas9, we repaired *HAP1* and found that SPD still exhibited toxicity in ρ^0^ cells, ruling out its involvement.

This observed ρ^0^-dependent SPD toxicity was not present in all strains examined. The ability of SPD to extend lifespan in D273-10B ρ^0^ cells demonstrates that this SPD toxicity is not a general phenomenon, but one that is controlled by genetic determinants. Toxicity of SPD in S288C ρ^0^ cells was expected as BY4741 is isogenic to S288C, aside from presence of mutated marker genes such as *ura3*. We also saw SPD toxicity in W303. This is a significant finding as 4 genes, *SAL1*, *CAT5*, *MKT1*, and *MIP1* have been linked to petite formation in S288C (Dimitrov et al. 2009). Three of these, *SAL1*, *CAT5*, and *MKT1*, have the same alleles in both BY4741 and W303 strains (Dimitrov et al. 2009), and can therefore be ruled out as a cause of the SPD toxicity we see. This is an interesting observation as it means that these two phenotypes of BY4741, high petite formation and SPD toxicity of ρ^0^ cells, must be separate phenomena. We have not formally ruled out Mip1p, the mtDNA polymerase. However, conceptually it is difficult to see a role of Mip1p in SPD toxicity in ρ^0^ cells as there is no mtDNA for it to act on. Nonetheless, we do not preclude an action of Mip1p beyond mtDNA replication and are planning to investigate this possibility in the future. With respect to D273-10B, this strain differs significantly from S288C. Future efforts will be to map and use genomic analyses to identify the genes or polymorphisms responsible for SPD toxicity in ρ^0^ cells.

The toxicity of SPD has been known since at least 1926 (Tabor and Rosenthal 1956). Aldehydes resulting from the oxidation of the amino group(s) may be a contributing factor (Pegg 2013). More recently, free SPD has been suggested to oxidize Fe^2+^ while concomitantly reducing O_2_ to the highly reactive O_2_^.-^ radical (Kumar et al. 2022). These studies were conducted in *Escherichia coli* at SPD doses (3.2–4.5 mM) similar to what was used in this study (4 mM). It is therefore possible that the SPD toxicity seen is due to the lack of mitochondrial respiration, resulting in elevated cellular O_2_ levels that can be more readily converted into O_2_^.^^-^. However, this explanation is difficult to rationalize as SPD extends lifespan in D273-10B ρ^0^ cells, despite these cells being more sensitive than BY4741 ρ^0^ cells to the O_2_^.^^-^ generator paraquat. Thus, our findings argue against SPD toxicity in ρ^0^ cells being due to the production of excess oxygen free radicals. Interestingly, it has been reported that SPD can protect yeast against heat stress through the induction of nitric oxide in a mitophagy-dependent manner (Kaur et al. 2021). This could, in principle, explain the SPD toxicity we see, as nitric oxide levels may be lower in ρ^0^ cells. However, the authors only see elevated spermidine levels at 37 °C and not at 30 °C, the temperature at which we conducted our experiments.

The ability of spermidine to extend lifespan has been proposed to involve the induction of autophagy (Eisenberg et al. 2009). Autophagy is the turnover of cellular components and is thought to be an important factor in lifespan extension due to dietary restriction (Aman et al. 2021). Under conditions of starvation, when cellular building blocks are in short supply, increasing turnover would free these building blocks to construct new cellular components. As a secondary effect, autophagy would remove damaged and defective cellular components such as proteins and mitochondria. With respect to autophagy-related gene expression, SPD showed a complex result. It appeared to down-regulate *ATG8*, which encodes a protein required for autophagy (Shpilka et al. 2011), and had no effect on *ATG33*, which is necessary for mitochondrial specific autophagy (Kanki et al. 2009). Yet, we found that SPD toxicity was absent in *ATG8*-deficient yeast cells, supporting a significant role of autophagy. Quite interestingly, SPD still exhibited toxicity in *ATG33*-deficient cells in which mitochondrial specific autophagy was disrupted. This is a perplexing finding as general autophagy appears to drive this toxicity, while the turnover of mitochondria, which are the dysfunctional organelles in the model we are using, does not. Overall, these results suggest that autophagy plays a role in the SPD toxicity in ρ^0^ cells, but additional factors must be involved.

Another reason for SPD toxicity is that autophagy is secondary to the depletion of ATP. When autophagy is induced in ρ^0^ cells, ATP levels are further depleted which results in such low levels that survival is compromised. This was supported by a direct measure of ATP in which its steady state levels are lowest in BY4741 cells treated with both SPD and EtBr. However, low ATP is an unlikely explanation because deletion of *ATG33* resulted in a 50-fold increase in steady-state ATP levels while still exhibiting SPD toxicity. Yeast mitochondria contain upwards of 2000 proteins and participate in many cellular processes such as fatty acid metabolism and amino acid synthesis. Another possibility explaining SPD toxicity is perhaps BY4741 has a lower total mitochondrial content than does D273-10B. The loss of mtDNA may further decrease mitochondrial content thereby impairing other cellular pathways. We found that BY4741 does indeed have lower mitochondrial content than does D273-10B and that the loss of mtDNA further lowers this. However, SPD had no effect on these values. Yet another possibility is that some cytoplasmic cellular process is being adversely affected. The acidification of the media due to the generation of acetic acid can be used as a crude marker of cellular metabolism (Murakami et al. 2011). We observed that the addition of SPD in ρ^0^ D273-10B cells elevated media pH to levels higher than in ρ^+^ cells and to levels approximating those seen in both ρ^+^ and ρ^0^ BY4741 cells fed SPD. This suggests that there is some metabolic pathway affected by SPD in D273-10B ρ^0^ cells, which is not present in BY4741 cells.

Loss of yeast mtDNA would result in the disruption of respiratory chain complexes III, IV, and V (Dujon 2020). We asked whether the impairment of the complexes individually would be sufficient to recreate this toxicity. The finding that only complex III was capable of recapitulating SPD toxicity demonstrates that loss of respiration and electron transport in general is not the cause. In addition, loss of complex V activity supports the idea that mitochondrial ATP is not the cause either. To evaluate the role of electron flow into the respiratory chain, we disrupted the yeast NADH dehydrogenase and succinate dehydrogenase (SDH) genes. SPD was able to extend lifespan even with the loss of the NADH dehydrogenases. Interestingly, SPD was not able to extend the lifespan of *SDH2*-deficient cells, though it was not toxic either. This demonstrates that SDH activity is essential for the lifespan-extending ability of SPD, as well as involved in the SPD toxicity we observed.

No matter the reason, the stark difference in the effect of SPD between yeast strains lacking mtDNA illustrates a particular caution that may be warranted for the use of SPD to slow aging in humans. The SPD toxicity we observed in BY4741 ρ^0^ cells appears to be a complex phenotype. It is clearly related to complex III, but also to autophagy, and complex II and the TCA cycle. If mitochondrial dysfunction is one of the important drivers of the aging process, the use of SPD could potentially harm the very individuals it is intended to help depending on their genetics. Consideration of these confounding factors in the consumption of SPD is particularly important as SPD is a readily available dietary supplement and can be purchased and used without medical supervision. In the future, we plan to expand our work to characterize this phenomenon in more strains and to use genomic analyses and gene mapping to identify the precise genes driving the phenotype.

## Supplementary Information

Below is the link to the electronic supplementary material.

Supplementary file1 (DOCX 305 kb)

## Data Availability

No datasets were generated or analysed during the current study.

## Abbreviations

SPD: Spermidine
EtBr: Ethidium bromide
CLS: Chronological lifespan
mtDNA: Mitochondrial DNA
ρ^+^: Yeast with mtDNA
ρ^0^: Yeast lacking mtDNA
KO: Knockout/deleted gene
PQ: Paraquat
