# Supplementary material for: Spermidine toxicity in Saccharomyces cerevisiae due to mitochondrial complex III deficiency
Source: Biogerontology. 2025 Apr 10;26(2):91. doi: 10.1007/s10522-025-10233-y (PMC11985560; doi:10.1007/s10522-025-10233-y)
Supplement: Supplementary file 1 — Supplementary file1 (DOCX 305 kb) [file 10522_2025_10233_MOESM1_ESM.docx]

**Supplementary data**


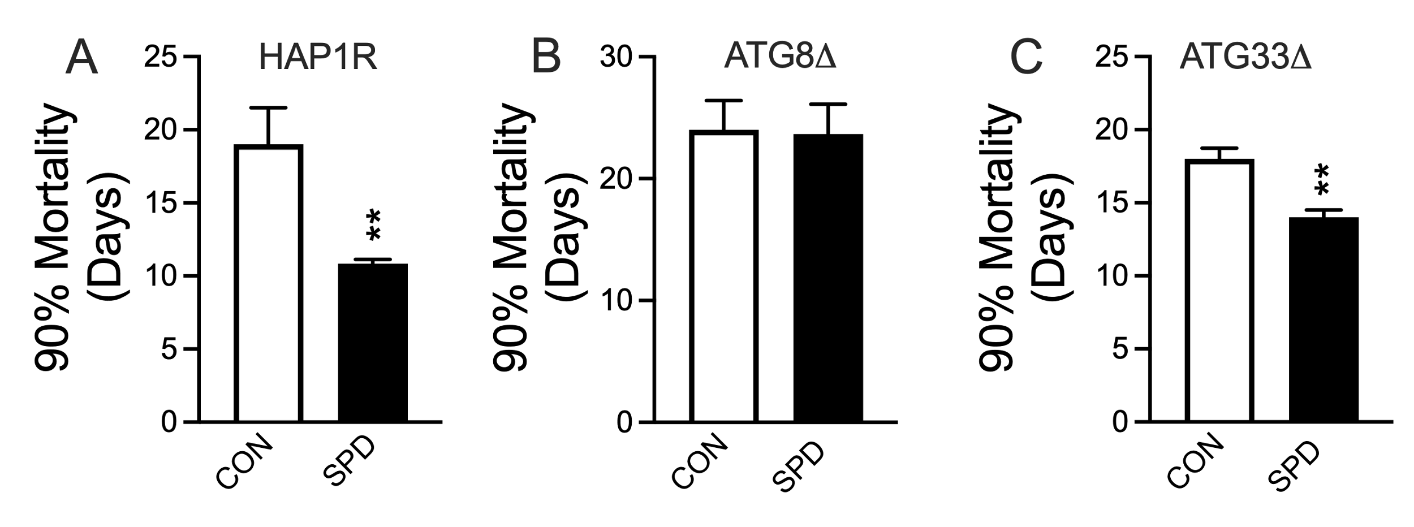


**Fig. S1**. The effect of SPD on the 90% mortality lifespan of **A** HAP1R cells, **B** ATG8 KO cells, and **C** ATG33 KO cells. ***P*<0.005, *P*=0.92 for ATG8Δ, unpaired t test. n=6 for each group.


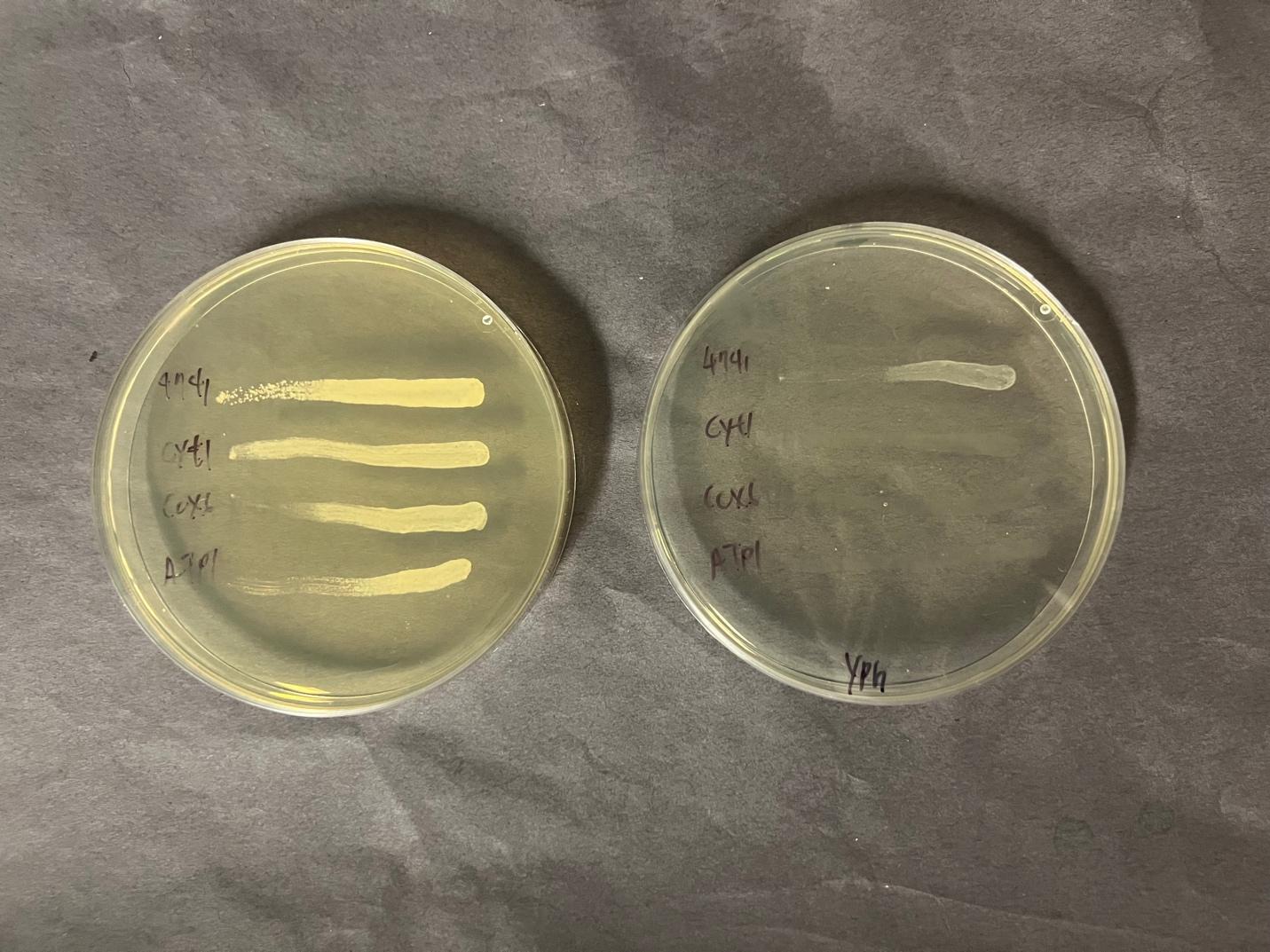


**Fig. S2**. Growth of BY4741 control cells and CYT1, COX6, and ATP1 deficient cells on glucose (left plate) and glycerol (right plate).

**Table S1**. Yeast strains used in this study

| Strain | genotype | source |
| --- | --- | --- |
| S288C | *MATα SUC2 gal2 mal2 mel flo1 flo8-1 hap1 ho bio1 bio6* | ATCC:204508 |
| W303-1B | *MAT*α *leu2-3,112 trp1-1 can1-100 ura3-1 ade2-1 his3-11,15* | ATCC:201238 |
| BY4741 | MATa his3Δ1 leu2Δ0 met15Δ0 ura3Δ0 | Horizon discovery |
| D273-10B | *MAT*α *mal GAL* | ATCC:24657 |
| BY4741 HAP1 repaired | MATa his3Δ1 leu2Δ0 met15Δ0 ura3Δ0 hap1Δ::HAP1 | This work |
| nde1Δ,BY4741 | MATa his3Δ1 leu2Δ0 met15Δ0 nde1::KanMX | Horizon discovery |
| nde1Δ,nde2Δ  BY4741 | MATa his3Δ1 leu2Δ0 met15Δ0 nde1Δ*::*KanMX,nde2Δ*::*URA3 | This work |
| ndi1Δ BY4741 | MATa his3Δ1 leu2Δ0 met15Δ0 ura3Δ0 nde1Δ*::*KanMX | Horizon discovery |
| sdh2Δ BY4741 | MATa his3Δ1 leu2Δ0 met15Δ0 ura3Δ0 sdh2Δ*::*KanMX | Horizon discovery |
| cyt1Δ BY4741 | MATa his3Δ1 leu2Δ0 met15Δ0 ura3Δ0 cyt1Δ*::*KanMX | Horizon discovery |
| cox6Δ BY4741 | MATa his3Δ1 leu2Δ0 met15Δ0 ura3Δ0 cox6Δ*::*KanMX | Horizon discovery |
| atp1Δ BY4741 | MATa his3Δ1 leu2Δ0 met15Δ0 ura3Δ0 atp1Δ*::*KanMX | Horizon discovery |
| atg8Δ BY4741 | MATa his3Δ1 leu2Δ0 met15Δ0 ura3Δ0 atg8Δ*::*KanMX | Horizon discovery |
| atg33Δ BY4741 | MATa his3Δ1 leu2Δ0 met15Δ0 ura3Δ0 atg33Δ*::*KanMX | Horizon discovery |

**Table S2**. Primers and oligos used in this study

| Primer/oligo | Sequence (5’ to 3’) |
| --- | --- |
| ACTF | TCGTTCCAATTTACGCTGGTT |
| ACTR | CGGCCAAATCGATTCTCAA |
| ATG7F | TTGCAATACGATGTTCCTGACTTGA |
| ATG7R | TGCTAGCTTACCTTGCACATTCCTT |
| ATG8F | TCGGAGAGGATTGCTGACAG |
| ATG8R | CTACGGTAAGGTCAGCAGGA |
| GAL1F | TGCTTTGTCAAATGGATCATATGG |
| GAL1R | CCTGGAACCAAGTGAACAGTACAA |
| COX1F | CACCACTAATTGAAAACCTFTCTG |
| COX1R | GATTTATCGTATGCTCATTTCCAA |
| MIP1F | GCATACCCAGACGAAGAGC |
| MIP1R | CAGTGCAGCAGGATCATCAC |
| CYT1F | TTACATCCCTGGCCCATACC |
| CYT1R | ACCACCGTGTCTAGCTTTCA |
| HAP1F | 5’-CAACGGCAGTGCTTTATCGA-3’ |
| HAP1R | GCGTTTCAGCTTCCACTAATT |
| HAP1 gRNAF | GATCATCAATTTCAACATTATCAAGTTTTAGAGCTAG |
| HAP1 gRNAR | CTAGCTCTAAAACTTGATAATGTTGAAATTGAT |
| NDE2F | TAAGTCAGCCCCTCCCTTTG |
| NDE2R | CGCGAATCTTCCATGACCAA3’ |
| NDE2 gRNAF | GATCGGTCATCTTGAAACGGTGTAGTTTTAGAGCTAG |
| NDE2 gRNAR | CTAGCTCTAAAACTACACCGTTTCAAGATGACC |
| HAP1  Repair template | CCTAATAACCTTGGAAGAAATGACGCGGTTGATTTTCTACC  AGTTGATAATGTTGAAATTGATGGACTAGTAGATTTTTATAGA  GCAGATTTTCCAATATGGGAGTGATGTTGGAATAAAAATCAACT  ATCATCTACTAACTAGTATT |
